# Clinical characteristics of Polish patients with molecularly confirmed Mowat-Wilson syndrome

**DOI:** 10.1007/s13353-021-00636-1

**Published:** 2021-05-12

**Authors:** Aleksandra Jakubiak, Krzysztof Szczałuba, Magdalena Badura-Stronka, Anna Kutkowska-Kaźmierczak, Anna Jakubiuk-Tomaszuk, Tatiana Chilarska, Jacek Pilch, Natalia Braun-Walicka, Jennifer Castaneda, Katarzyna Wołyńska, Marzena Wiśniewska, Monika Kugaudo, Monika Bielecka, Karolina Pesz, Jolanta Wierzba, Anna Latos-Bieleńska, Ewa Obersztyn, Małgorzata Krajewska-Walasek, Robert Śmigiel

**Affiliations:** 1 Department of Paediatrics, Division of Paediatric Propaedeutics and Rare Disorders, Medical University, Wroclaw, Poland; 2 Department of Medical Genetics, Medical University, Warsaw, Poland; 3 Department of Medical Genetics, Medical University, Poznan, Poland; 4 Department of Medical Genetics, Institute of Mother and Child, Warsaw, Poland; 5 Department of Neurology and Children Rehabilitation, Medical University, Bialystok, Poland; 6 Medical Genetics Unit, Mastermed Medical Center, Bialystok, Poland; 7 Department of Genetics, Polish Mother’s Memorial Hospital Research Institute, Lodz, Poland; 8 Department of Paediatric Neurology, Medical University of Silesia, Katowice, Poland; 9 Department of Children and Adolescent Psychiatry, University Clinical Centre, Paediatric Teaching Clinical Hospital, Warsaw, Poland; 10 Department of Pharmaceutical Biotechnology, Medical University, Wroclaw, Poland; 11 Department of Genetics, Medical University, Wroclaw, Poland; 12 Department of Internal and Paediatric Nursing, Institute of Nursing and Midwifery, Medical University Gdansk, Gdansk, Poland; 13 Department of Medical Genetics, The Children’s Memorial Health Institute, Warsaw, Poland

**Keywords:** Mowat-Wilson syndrome, Phenotype, Dysmorphism, Congenital anomalies, *ZEB2* gene

## Abstract

Mowat-Wilson syndrome is a rare neurodevelopmental disorder caused by pathogenic variants in the *ZEB2* gene, intragenic deletions of the *ZEB2* gene, and microdeletions in the critical chromosomal region 2q22-23, where the *ZEB2* gene is located. Mowat-Wilson syndrome is characterized by typical facial features that change with the age, severe developmental delay with intellectual disability, and multiple congenital abnormalities. The authors describe the clinical and genetic aspects of 28th patients with Mowat-Wilson syndrome diagnosed in Poland. Characteristic dysmorphic features, psychomotor retardation, intellectual disability, and congenital anomalies were present in all cases. The incidence of most common congenital anomalies (heart defect, Hirschsprung disease, brain defects) was similar to presented in literature. Epilepsy was less common compared to previously reported cases. Although the spectrum of disorders in patients with Mowat-Wilson syndrome is wide, knowledge of characteristic dysmorphic features awareness of accompanying abnormalities, especially intellectual disability, improves detection of the syndrome.

## Introduction

Mowat-Wilson syndrome (MWS, OMIM 235,730) is a rare congenital disorder characterized by developmental delay with subsequent severe intellectual disability, distinctive facial features, and characteristic congenital anomalies. The patients have severe speech delay, and language is usually restricted to few words. The behavioral phenotype is similar to that found in Angelman syndrome. Individuals with MWS seem happy and easy-going. The phenotype includes also stereotypical movements and various congenital malformations, such as Hirschsprung disease, heart defects, corpus callosum agenesis, genitourinary system anomalies, and eye abnormalities. Facial dysmorphism in MWS becomes unique as it evolves with age.

Birth incidence of MWS is currently unknown and possibly underestimated at 1–2/100,000. The pathogenic variants in the *ZEB2* (*ZFHX1B*) gene, intragenic deletions, and microdeletions of the critical region 2q22-23, in which the *ZEB2* gene is located, all cause Mowat-Wilson syndrome. Many *ZEB2* variants have been described with no apparent mutational hotspots. The only genotype–phenotype correlation is based on differences in clinical course of disease in patients with point mutations in the *ZEB2* gene and deletions comprising the critical region 2q22-23.

We report on clinical and genetic findings of 28 Polish patients with a diagnosis of MWS.

## Material and methods

A working diagnosis of Mowat-Wilson syndrome was established based on clinical features: facial dysmorphism, developmental delay or intellectual disability, and congenital anomalies, including heart defects, hypospadias, cryptorchidism, brain defects, and Hirschsprung disease. All patients had a normal karyotype. In some patients, other cytogenetic and molecular studies were performed (methylation test for Angelman syndrome, FISH study for the 22q11.2 region, MLPA telomere probemix, array-CGH) and proved to be normal.

To confirm Mowat-Wilson syndrome’s diagnosis, we performed molecular tests, including direct sequencing and MLPA analysis of the *ZEB2* gene, and eventually aCGH. One patient has been already reported (11).

Direct bidirectional sequencing of *ZEB2* was performed after PCR amplification of all coding exons (2–10) and the noncoding exon 1 and analyzed using an automated capillary sequencer (ABI 3730, Applied Biosystems Corp.). Sequences were analyzed with the SeqPilot (JSI medical systems GmbH) and compared to the reference sequence NCBI35:17:19,492,183:19,521,995:1. MLPA reaction of the patients’ DNA was performed with the P169 Kit by MRC Holland according to the supplier’s instructions. The analysis was performed with the ABI 3100 capillary sequencer (Applied Biosystems Corp.) and the software SeqPilot (version 2.0) by JSI medical systems GmbH. In one case, the diagnosis was confirmed by the oligonucleotide-array-CGH analysis, performed by kit 8 × 60 K by Agilent.

## Results

Molecular tests confirmed Mowat-Wilson syndrome in all presented cases. In twenty-one patients, we identified a point mutation (75%). Two patients had the same point mutation (c.607insTGGA). We found exonic deletions in the *ZEB2* gene in six patients (21%), including the deletion of all exons (1–10) of the gene in two cases. By array-CGH, we identified a large deletion of 8 Mb in the 2q22.3q23.3 region, including the entire *ZEB2* gene in one patient (Table 1).

**Table 1:** Clinical characteristics of our patients

No	Sex	Mutation in ZEB2 gene	ID/DD	Speech	Cardiac defect	Hirschsprung disease/constipation	Brain defect	Cryptorchid testes/hypospadias in males	Renal defect	Epilepsy	Other	Death, age
1	F	c.1027C > T (p.R343*)	+	No	-	-	Hypoplasia of corpus callosum	Not applicable	Hydronephrosis	-	Axenfeld anomaly	-
2	F	Deletion ex. 1–10	+	No	ToF	HD	ACC	Not applicable	Hydronephrosis	-	-	3y + due to complications of Hirschsprung disease
3	F	c.648C > A (p.C216*)	+	No	Anomaly Ebstein	Constipation	Ventriculomegaly	Not applicable	Vesicoureteric reflux	+	-	-
4	F	Deletion ex. 10	+	No	ASD	Constipation	-	Not applicable	-	-	Hypo and hyperpigmentation	-
5	F	c.1946delT (p.I649Tfs*17)	+	No	ASD,VSD	-	-	Not applicable	-	+	-	-
6	M	c.607insTGGA (p.Thr203IlefsTer37)	+	No	VSD	Constipation	ACC	Cryptorchidism	-	+	-	Rhabdomyosarcoma of the head, nasal sinus and orbit region
7	M	c.399_400dupTA (p.Thr134IlefsTer3)	+	No	-	-	ACC	Cryptorchidism	-	-	-	-
8	M	c.1276 T > A (p.Leu426Ile)	+	No	-	Constipation	Ventriculomegal, cortical atrophy	Cryptorchidism	-	-	Myopia	-
9	M	c.696C > G (p.Y232*)	+	No	ToF	Constipation	-	-	-	-	Accessory nipples	-
10	M	Deletion 8 Mb, 2q22.3q23.3 (aCGH)	+	No	DORV, HLHS, VSD,ASD	HD	ACC	Cryptorchidism	-	-	Microphthalmia	6 m + , heart defect
11	M	Deletion ex. 1–10	+	No	ToF	HD	ACC	Hypospadias cryptorchidism	-	+	Iris coloboma	-
12	M	Deletion ex. 3–10	+	No	ToF, pulmonary stenosis	-	Cortical atrophy	Hypospadias	+	-	Iris coloboma	-
13	F	c.857-858delAG (p.Glu286ValfsTer8)	+	No	VSD, PDA aortic coarctation	HD	ACC	Not applicable	Hydronephrosis	-	Iris coloboma	-
14	M	c.607insTGGA (p.Thr203IlefsTer37)	+	No	VSD, pulmonary stenosis	Constipation	Hypoplasia of corpus callosum	Cryptorchidism, hypospadias	-	+	Accessory nipples, cancer	-
15	M	c.1445 T > G, (p.Leu482*)	+	No	-	Constipation	-	-	Vesicoureteric reflux, hydronephrosis, posterior urethral valves	-	-	-
16	F	c.84 T > G (p.Tyr28*)	+	No	Bicuspid aortic valve	**-**	-	Not applicable	-	+	Astigmatism, irides heterochromia	-
17	F	c.1421-1426delA (p.Gln474_Met476delinsLeu)	+	No	VSD,ASD	Constipation	Ventriculomegaly	Not applicable	-	-	-	-
18	M	c.2230A > G (p.Ile744Val)	+	No	-	HD	ACC	Cryptorchidism	+	+	Microphthalmia	-
19	M	c.3202G > T (p.Gly1068Cys)	Mild	Single words	-	Constipation	-	Cryptorchidism	-	-	-	-
20	F	c.2087_2088del (Lys696Serfs*24)	Severe	No	VSD	HD	Hypoplasia of corpus callosum	Not applicable	-	+	-	-
21	M	c.2073G > A (p.Trp691Ter)	Moderate	No	ASD	Constipation	ACC	Cryptorchidism	-	+	Liver hemangioma, strabismus	-
22	M	Deletion ex. 2–9	+	No	Bicuspid aortic valve	HD	Cortical atrophy, ventriculomegaly	Cryptorchidism	-	+	-	-
23	F	c.2562_2564delAA (p.N855Lfs*3)	Severe	No	-	Constipation	-	Not applicable	-	+	Sacral dimple	-
24	F	c.1177dupG (p.E393Gfs*7)	Severe	No	-	HD	-	Not applicable	-	+	Bilateral cataracts	-
25	F	c.1437_1440delCA (p.H304Qfs*3)	+	No	PDA, VSD	Constipation	-	Not applicable	-	+	Pale optic discs,	-
26	F	c.2083 C > T (p.Arg695Ter)	+	No	PDA	-	Hypoplasia of corpus callosum	Not applicable	-	+	Myopia	-
27	M	c.2083C > T (p.Arg695Ter)	Mild	No	AVSD, pulmonary stenosis, bicuspid aortic valve	-	ACC, colpocepaly	Not applicable	-	-	Enlarged spleen with multiple small cysts	-
28	F	Deletion ex. 3–10 aCGH 258,2 kbp	Severe	No	VSD	-	ACC	Hypospadias cryptorchidism	Vesicoureteric reflux, urolithiasis	+	Submucosal cleft palate	-

*ID* intellectual disability, *DD* developmental delay, *HD* Hirschsprung disease, *ACC* agenesis of corpus callosum, *ASD* atrial septal defect, *VSD* ventricular septal defect, *AVSD* atrioventricular septal defect, *ToF* tetralogy of Fallot, *DORV* double outlet right ventricle, *HLHS* hypoplastic left heart syndrome

MWS was diagnosed in 14 females and 14 males raging in ages from 3 months to 28 years. All patients had a developmental delay or intellectual disability (in 27 cases, it was moderate or severe; only one patient presented with a mild degree). Only one patient (with mild intellectual disability) could speak single words; in the remaining patients, speech was absent (in those older patients, perception of speech is much better than expressive language). All children had at least one congenital anomaly. Among the most common, there were cardiac defects (20/28), brain defects (19/28, Hirschsprung disease (8/28), and genital anomalies in males (12/14). Cardiac defects included the following: tetralogy of Fallot (4/20), Ebstein anomaly (1/20), atrial septal defect (6/20), ventricular septal defect (9/20), pulmonary stenosis (3/20), bicuspid aortic valve (3/20), double outlet right ventricle, and hypoplastic left heart syndrome (1/20) and atrioventricular septal defect (1/20). Eight children had more than one cardiac anomaly. The most prevalent brain malformation was agenesis/hypoplasia of corpus callosum (14/19); the remainder included the following: ventriculomegaly (4/19), cortical atrophy (2/19), and colpocephaly (1/19). Fifteen patients had epilepsy or abnormal EEG pattern despite anti-epileptic treatment (54%). Hirschsprung disease was presented in 8 patients and always correlated with constipation, but 12 patients had constipation without Hirschsprung disease. The females in this group did not have any genital defects. Among genital defects in males, undescended testicles and hypospadias were found in 8/14 and in 1/13, respectively. Three male patients had both these defects. Anomalies of the urinary tract, such as hydronephrosis, vesicoureteral reflux, and posterior urethral valve and urolithiasis, were present in 29% (8/28). Other notified abnormalities were as follows: ocular defects ((Axenfeld anomaly (1/28), iris coloboma (3/28), high myopia (2/28), strabismus (1/28), astigmatism (1/28), bilateral cataracts (1/28), pale optic disc (1/28), heterochromia of iris (1/28), microphthalmia (2/28)), liver hemangioma (1/28), accessory nipples (2/28), hypo and hyperpigmentation of the skin(1/28), submucosal cleft palate (1/28), enlarged spleen with multiple small cysts (1/28), and rhabdomyosarcoma with fatal course of disease (1/28).

**Fig. 1:**
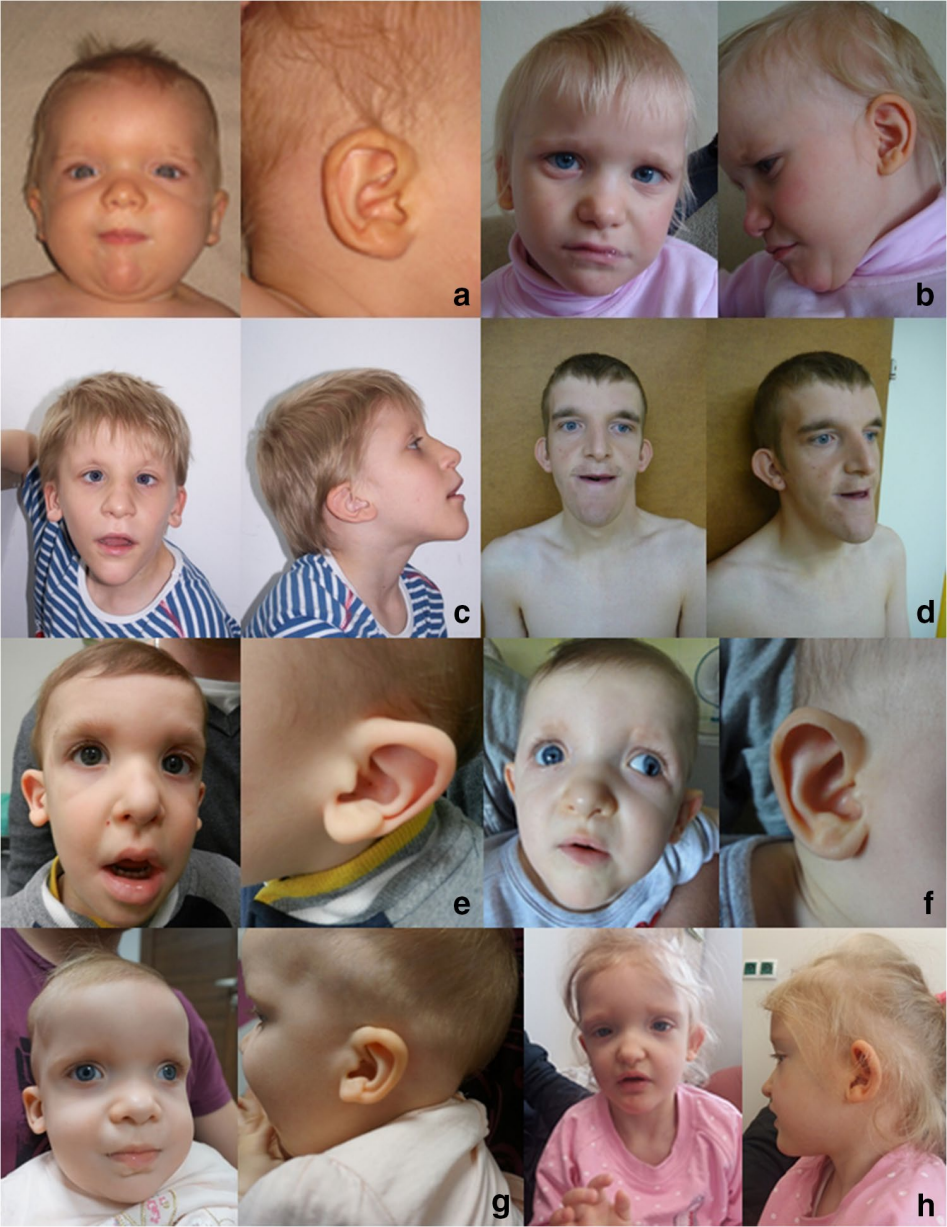
Facial features of patients with Mowat-Wilson syndrome, frontal and profile views. A patient 1, B patient 2, C patient 6, D patient 7, E patient 12, F patient 13, G patient 20, H patient 23

All diagnosed patients had characteristic facial dysmorphic features that evolved with age. A square-shaped face in young children that becomes elongated with age, hypertelorism, prominent ear lobes with central depression on the outer surface, and pointed or triangular chin are the most striking features. In some patients, prognathism, short nose, becoming longer with age, with a broad base, bulbous, upturned nasal tip, and short columella (broad in older children and adults), epicanthic folds, and open mouth were observed (Fig. 1).

The children were microcephalic (below 3rd percentile and below − 2 SD) with the weight below the 10th percentile and height less than 25 percentile.

Another feature that found to be quite distinctive is behavior: the children seem happy and cheerful with an open personality and stereotypical movements of hands.

Three patients had died: one due to Hirschsprung disease complications; one due to rhabdomyosarcoma of the nasal sinus, and orbital region; one patient due to a complex cardiac defect (DORV, HLHS, VSD, ASD).

## Discussion

Mowat-Wilson syndrome is a sporadic congenital disorder. David Mowat and Meredith Wilson described the condition as a separate entity for the first time in 1997 at the Clinical Genetics Department, Children’s Hospital at Westmead, Sydney. The patients described in the original paper presented with an intellectual disability, specific facial dysmorphism, and Hirschsprung disease (Mowat et al. 1998). These features seemed constant in the following papers reporting on individuals with MWS. Initially, Hirschsprung disease was considered a necessary feature of the syndrome. Later, clinicians revealed that Hirschsprung disease is not required for the diagnosis (Zweier et al. 2002, Garavelli and Mainardi 2007).

We report on 28 cases of MWS diagnosed in Poland. The first patient was previously described (Smigiel et al. 2015), but the others are presented for the first time. All patients have the classical phenotype, intellectual disability, and various congenital anomalies. Table 1 summarizes the clinical findings observed in our patients.

The distinctive facial appearance was reported in all cases described in the literature (Garavelli and Mainardi 2007, Ivanovski et al. 2018). Dysmorphic features change with age (Zweier et al. 2002, Ivanovski et al. 2018). Infants have a round or square face with a small, pointed chin. Later, the face becomes elongated with marked prognathism. Eyes are large and deeply seated, with hypertelorism or telecanthus. Eyebrows are prominent, and with age, they may become heavier, with medial flare. Ears may be low set and posteriorly rotated, with very unique large, fleshy, and uplifted earlobes, with central depression. Ears do not change with age, but the central depression on earlobes may become less prominent. The nose is relatively small in infants, with a broad root; later, it becomes longer with a low set tip and pronounced columella (Zweier et al. 2003, Ivanovski et al. 2018, Ho et al. 2020). Dental anomalies, such as widely spaced teeth, dental crowding, malposition of teeth, and delayed tooth eruption, may be present. In 82% of cases, microcephaly was described. It may be present at birth, but usually, it develops throughout infancy. Birth weight and length generally are within a normal range. Later in life, patients have short stature (length or height 2 SD below the mean); the mean adult height is 165.1 in males and 150.5 cm in females. Also, weight might be decreased; it is below the third percentile in 30% of patients (Ivanovski et al. 2018). Apart from developmental delay and subsequent intellectual disability, facial dysmorphism was one of the main criteria that suggested the diagnosis of MWS in our patients (Fig. 1).

Except dysmorphism, the constant feature present in all patients is developmental delay and subsequent intellectual disability, usually moderate to severe (Zweier et al. 2002, Ivanovski et al. 2018). Language development is severely impaired in MWS patients. Some do not develop expressive language, whereas receptive language is more advanced (Ivanovski et al. 2018, Ho et al. 2020). In our cohort, only one child developed an active language. Gross motor skills are also delayed, the mean age of walking is 3.5 years, but some patients do not achieve ambulation. Gait may be impaired similarly to Angelman syndrome—wide-based, ataxic with flexed arm position (Ishihara et al. 2004, Ho et al. 2020). A common finding in patients with MWS is epilepsy. In our group, 54% of patients experienced seizures, which is less compared to other reports (70–75%) (Cordelli 2013a). Cordelli et al. (2013a, b) described the most typical seizures in patients with MWS as frontal lobe and atypical absence seizures. They are often preceded by fever‐triggered seizures (Cordelli 2013a).

The personality of patients with MWS is usually cheerful and sociable. It may resemble the behavior in Angelman syndrome, with frequent smiling and laughter. Some patients have stereotypical movements, repetitive behaviors, and high pain tolerance (Evans et al. 2012, Ishihara et al. 2004, Ivanovski et al. 2018).

Congenital malformations are common findings in patients with MWS. In our cohort, all patients have at least one malformation. In the first descriptions in literature, all patients had Hirschsprung disease, which was considered a necessary feature for the diagnosis (Mowat et al. 2003, Mowat et al. 1998). Later research showed that Hirschsprung disease is present in about 50% of patients. Constipation, without Hirschsprung disease, is present in about 30% of cases (Zweier et al. 2002, Zweier et al. 2005). In our cohort, eight patients had Hirschsprung disease; constipation without Hirschsprung disease was reported in 12 patients (43%). One patient had constipation and atypical defect of the colon, not connected to Hirschsprung disease.

Variable heart defects are common among patients with MWS. They are found in 50–80% of cases and affect all heart structures. They are commonly not complex and include defects of pulmonary arteries and valves, septal defects, patent ductus arteriosus, and tetralogy of Fallot and aortic coarctation (Ishihara et al. 2004, Ivanovski et al. 2018). In the presented group, 71% of patients had heart defects. The most common was VSD, usually cooccurring with other defects (such as ASD or pulmonary stenosis).

Hypogenesis and agenesis of the corpus callosum are the most common brain anomalies in patients with MWS. In this series, they were almost as frequent as in the literature (50% in our series vs. 46% in the literature). Seven patients had other brain abnormalities. According to literature, less common brain anomalies include cortical atrophy, ventriculomegaly, pachygyria, cerebellar hypoplasia, the hippocampus’ defects, and frontotemporal atrophy (Ishihara et al. 2004, Ivanovski et al. 2018, Ho et al. 2020).

Genitourinary defects are common in patients with MWS; they are present in about 50% of cases (Ivanovski et al. 2018). In our series, 51% of patients had urogenital or renal anomalies. Eleven (79%) male patients had cryptorchidism, which is significantly more than in literature, and only four of them had hypospadias.

Usually, hearing is normal; up to date, only two patients were reported to have sensorineural deafness. More often, the patients suffer from recurrent otitis media (Ishihara et al. 2004, Ivanovski et al. 2018). In our cohort, none of the children experienced a hearing deficit.

Strabismus is a frequent finding in patients with MWS. Nystagmus may be present in infancy, but it usually resolves with age. Other ophthalmologic findings include myopia, astigmatism, microphthalmia, colobomas, Axenfield anomalies, and cataract (Ivanovski et al. 2018, Ho et al. 2020). In our cohort, strabismus was rare (found in only one patient), which is quite unusual. Coloboma was the most frequent ophthalmological finding.

Several skeletal anomalies were described in MWS patients, such as chest anomalies (three patients in our cohort), feet anomalies ((pes planus, calcaneal-valgus deformity, anomalies of halluces)—five patients in our cohort), hands anomalies (abnormalities of thumbs, syndactyly, camptodactyly, ulnar deviation), and scoliosis (six patients in our cohort). The habitus is usually described as slender; some of the individuals have short stature. Later in life, interphalangeal joints may become more prominent (one patient in our group) (Ishihara et al. 2004, Ivanovski et al. 2018, Ivanovski et al. 2020).

Skin abnormalities are not very common but may be present, like accessory nipples or skin pigmentation defects (Zweier et al. 2003, Ishihara et al. 2004).

In 2013, Valera et al. described a case with medulloblastoma and glioblastoma in one patient with MWS. Subsequently, in 2017, Rogac et al. presented a girl with MWS and rhabdomyosarcoma and suggested an association between MWS and tumor development. One of our patients died due to rhabdomyosarcoma. However, the incidence of cancer in patients with MWS is low and probably does not exceed the population risk.

The only known cause of MWS is mutation in ZEB2 gene (Wakamatsu et al. 2001, Cacheux et al. 2001, Dastot-Le Moal et al. 2007). Up to date, different mutations (pathogenic variants in the *ZEB2* gene, intragenic deletions, and microdeletions of the critical region 2q22-23) were described. The majority of MWS cases are sporadic. Few familial cases may be due to gonadal or somatic mosaicism (Ho et al. 2020). Likewise, in our report, all cases were sporadic. The *ZEB2* gene’s haploinsufficiency caused all cases described in the literature with molecular confirmation of MWS. Usually, in patients with classic MWS, loss-of-function variants are reported. Missense variants in the *ZEB2* gene may be associated with a less severe phenotype (Ivanovski et al. 2018). Our patients have different pathogenic variants of the *ZEB2* gene; point mutations were more common than exon deletions. One patient with a large deletion found on aCGH (8 Mb) had a severe heart defect and died at 6 months of age. Both patients with c.607insTGGA mutation held similar phenotypes: except for dysmorphism and intellectual disability, they had heart defects (VSD, two-cuspid mitral valve, constipation without Hirschsprung disease, agenesis of the corpus callosum, cryptorchidism, hypospadias, accessory nipples, and epilepsy). Patients with a deletion of the whole *ZEB2* gene also had similar abnormalities (tetralogy of Fallot, agenesis of the corpus callosum, Hirschsprung disease). Phenotype-genotype correlations in the literature suggest that large deletions result in a more severe phenotype, while pathogenic variants that preserve some gene function give fewer complications. However, the prevalence of particular mutations is too low to establish a connection between mutation and phenotype (Zweier et al. 2003, Zweier et al. 2005, Ivanovski et al. 2018).

## Conclusion

Our paper is the first presentation of a large cohort of Polish MWS patients. There are not many differences between patients of other ethnicities. The presence of rare neoplasms such as rhabdomyosarcoma raises suspicion of a connection between *ZEB2* impairment and this type of cancer. However, there is still no evidence that there is a high risk of malignancy in MWS.

While MWS is a rare genetic syndrome with a variety of different symptoms, some are unique and quite specific to MWS. Distinctive facial gestalt in patients with developmental delay or intellectual disability should suggest the diagnosis of MWS even in the absence of congenital anomalies, seizures, and Hirschsprung disease.

## Data Availability

The authors affirm that this manuscript is an honest, accurate, and transparent account of the study being reported; that no important aspects of the study have been omitted; and that any discrepancies from the study as planned (and, if relevant, registered) have been explained.
